# Pseudorabies virus infection reshapes the host epitranscriptome by globally suppressing but selectively elevating specific RNA modifications

**DOI:** 10.3389/fmicb.2026.1790077

**Published:** 2026-03-27

**Authors:** Yuanyuan Wang, Qingsen Wang, Jiajia Zhong, Fanghui Mao, Xuyan Liu, Fanfeng Meng, Xiaoyong Chen

**Affiliations:** 1 Xingzhi College, Zhejiang Normal University, Jinhua, China; 2 Institute of Animal Husbandry and Veterinary Medicine, Fujian Academy of Agriculture Sciences, Fuzhou, China; 3 Zhejiang Combiwell Health Products Technology Development Co., Ltd., Jinhua, China; 4 Chia Tai Animal Husbandry Investment Co., Ltd., Beijing, China

**Keywords:** epitranscriptome analysis, pseudorabies virus, RNA modification, viral infection, virus-host interaction

## Abstract

RNA modifications are ubiquitous in host cells and hold crucial roles in regulating diverse biological processes. Although the m6A modification during viral infections has garnered significant attention, the involvement of other modifications in such infections remains underexplored. This study was aimed to map out the comprehensive RNA modification profiles in porcine kidney PK-15 cells infected with pseudorabies virus (PRV) at various time points. Throughout the PRV infection, we identified a total of 31 distinct RNA modifications, among which 25 were detected in significantly higher abundances. Our findings indicated that PRV infection largely suppressed the overall level of RNA modifications, but interestingly, it elevated specific modifications, namely Am, Cm, Gm, and Um. Upon deeper investigation, we discovered that PRV infection notably diminished the levels of enzymes related to m1A modification. Collectively, this study uncovers the landscapes of RNA modification in PRV infection, paving the way for further explorations into RNA modifications during herpesvirus infections.

## Introduction

Pseudorabies virus (PRV), an *alpha-herpesvirus*, serves as the causative agent of Aujeszky’s disease. This virus poses a significant threat to a diverse range of species, including humans, pigs, dogs, rabbits, rats, and mice (Chen et al., 2022). Among these, pigs serve as the natural reservoir for PRV, making them particularly vulnerable to its deleterious effects. PRV infection in pigs can lead to a wide array of debilitating symptoms, ranging from neurological disorders and respiratory issues to miscarriages in pregnant sows and even the death of piglets. These consequences not only inflict immense suffering on the animals but also result in considerable financial losses for the pig industry (Tan et al., 2021). In China, the Bartha strain vaccination has historically been employed as a strategy to combat PRV. However, towards the end of 2011, variants of PRV began to emerge and spread within the country. These new strains displayed altered characteristics after infecting pigs, complicating efforts to control the virus (An et al., 2013). Indeed, outbreaks of PRV became apparent in several large-scale pig farms despite their prior vaccination with the Bartha strain (An et al., 2013; Tong et al., 2015). Notably, in 2017, China reported the first incidence of human endophthalmitis caused by PRV infection (Ai et al., 2018). This revelation underscored the evolving nature of the virus and its potential to pose a threat to human health. Thus, there have been an increasing number of researches focusing on PRV, since the findings in 2020 that PRV infection may also result in acute encephalitis in people. Understanding the pathogenic mechanism of PRV and analyzing the alterations in multiple biological signal pathways in host cells after PRV infection are critical for scientifically preventing and controlling the PRV pandemic.

The discovery of a fifth nucleotide after studying yeast soluble RNA 60 years ago gave rise to the idea of a modified RNA nucleoside (Zhang et al., 2023). Then, in the 1970s, researchers found N6-methyladenosine, or m6A, existed in a variety of cellular mRNAs as well as in the mRNAs of viruses (Brocard et al., 2017). More than 100 different chemical alterations to RNA have been identified so far, the majority of which are shown to be prevalent in non-coding RNA, including tRNA, rRNA, and snRNA. They are crucial for sustaining their activities in translation and splicing (Machnicka et al., 2013). In short, RNA modifications highlight the importance of RNA epigenetics or the epitranscriptome. In addition to m6A, several other key modifications also play regulatory roles in gene expression, including 5-methylcytosine (5mC) and its oxidative derivatives, pseudouridine, 5-methylcytidine (m5C), and N1-methyladenosine (m1A) (Chen et al., 2016). In general, methyltransferases, or writers, and demethylases, or erasers, dynamically control modifications. These enzymes perform their functions by either directly recognizing binding proteins, or readers, or indirectly by adjusting the structure of the modified RNA to control RNA reader-protein interactions.

While it is known that some viral RNA, such as influenza virus (Li H. et al., 2022), Rous sarcoma virus (Kane and Beemon, 1985), and simian virus 40 (Tsai et al., 2018), contains m6A modifications, it has long been unclear how these modifications work and whether viruses affect the cellular m6A landscape. It is only recently shown that the connections between m6A alteration and viruses are starting to be understood based on the advent of genome-wide technologies to map m6A residues. Intriguingly, human immunodeficiency virus 1 (HIV-1) genomic RNA (gRNA) alterations localized in the U5/TAR and gag region play a surprising post-entry modulation function in infected Jurkat and primary CD4^+^ T cells (Tirumuru et al., 2016). Mechanistic analysis further connected this site’s recognition by cellular YTHDF1-3 proteins to a suppression of the reverse transcription of the viral gRNA, pointing to a cellular anti-viral mechanism that could account for variations in infectivity levels between laboratory strains and cultivable models (Tirumuru et al., 2016). Most recently, it was shown that the pseudorabies virus take advantage of the m6A alteration to enhance viral proliferation in pig kidney PK-15 cells (Yu et al., 2023). Beyond the previously understood mechanisms, the dynamic regulation of the epitranscriptome has recently emerged as a crucial layer of virus-host interactions in PRV and other animal herpesviruses. For instance, recent studies reveal that PRV can actively manipulate the host’s m6A machinery, such as through the UL13 protein kinase-mediated phosphorylation of the RNA demethylase FTO, to suppress interferon-stimulated gene expression and evade innate immunity (Verhamme et al., 2025). Furthermore, in other agriculturally significant animal herpesviruses, such as Marek’s disease virus (MDV), epitranscriptomic features like m6A modifications have been shown to colocalize with G-quadruplex structures to critically regulate viral replication and oncogenic gene expression (Yao et al., 2026). Exploring these conserved and diverse RNA modification strategies across animal herpesviruses provides a necessary and critical context for understanding how these pathogens broadly manipulate host cellular machinery (Verhamme and Favoreel, 2025). However, the impact of m1A alteration on viruses, particularly PRV, remains yet unknown.

In this study, we employed metabolomics techniques to gain a profound understanding of the global RNA modifications occurring in PK-15 cells infected with PRV at various time intervals. Our findings have not only revealed the intricate relationship between RNA modifications and the virus, but also illuminated the diverse ways in which they mutually influence each other. Moreover, we investigated the association between (m1A) modification and PRV infection. These insights offer a novel perspective on the pathogenicity of PRV and provide fresh understanding into the intricate interactions between the host and the virus.

## Materials and methods

### Cell line

Porcine kidney PK-15 cells (CCL-33, ATCC) were maintained in Dulbecco’s modified Eagle’s medium (DMEM, Bioland) supplemented with 10% fetal bovine serum (FBS, NEST Biotechnology), 100 U/mL penicillin, and 100 μg/mL streptomycin (Biolight) and treated at 37 °C under 5% CO_2_.

### Virus and virus infection

PRV RA strain was preserved in our laboratory (Chen X. et al., 2023). When PK-15 cells cultured in 6-well plates (CellPro Biotechnology) were grown to approximately 90% confluence, they were infected with PRV at a multiplicity of infection (MOI) of 1. After incubation for 1 h at 37 °C, the unattached viruses were washed three times using PBS. The cells were then maintained in DMEM supplemented with 2% FBS at 37 °C for different periods of time before collection. To determine the viral titers, the collected samples were serially diluted tenfold and inoculated onto PK-15 cells seeded in 96-well plates (A-GEN). The viral titers were analyzed using Kaerber’s method and exhibited as the 50% tissue culture infectious dose (TCID_50_) per milliliter.

### Sample preparation and RNA digestion

PK-15 cells were collected at 0 h (serving as the baseline mock-infected, virus-free control group), 16 h, 32 h, and 48 hpi, with three biological replicates in each group and more than 10^7^ cells in each replicate. Total RNA was extracted using Trizol reagent (KeygenBioTECH) at a ratio of 1 mL Trizol per 1 * 10^7^ cells. Following complete cell lysis in 1.5 mL EP tubes [BaiDi Biotechnology Co., Ltd. (BDBIO)], 200 μL of chloroform was added per 1 mL of Trizol. The mixture was vigorously shaken for 15 s, incubated at room temperature for 5 min, and then centrifuged at 12,000×*g* for 15 min at 4 °C to separate the aqueous phase. To ensure the RNA quality met the strict requirements for subsequent LC-MS/MS and qRT-PCR analyses, RNA quantity and purity were evaluated using a NanoDrop spectrophotometer. Only samples exhibiting an A260/A280 ratio between 1.8 and 2.0, and an A260/A230 ratio >2.0, were subjected to downstream RNA digestion. 2 μg of RNA was mixed with buffer, S1 nuclease, alkaline phosphatase, and phosphodiesterase I, following incubation at 37 °C. The mixture was extracted with chloroform once the RNA had entirely been digested into nucleosides. The resultant aqueous layer was collected for UPLC-ESI-MS/MS analysis.

### UPLC and ESI-MS/MS conditions

An UPLC-ESI-MS/MS system (UPLC, ExionLCTM AD; MS, Applied Biosystems 6,500 Triple Quadrupole) was used to analyze the nucleosides. The following analytical conditions applied: Waters ACQUITY UPLC HSS T3 C18 (1.8 m, 2.1 mm * 100 mm) column; water (2 mM NH_4_HCO_3_): methanol; gradient program; flow rate; temperature; and injection volume; 95:5 V/V at 0 min, 95:5 V/V at 1 min, 5:95 V/V at 9 min, 5:95 V/V at 11 min, 95:5 V/V at 14 min. An alternate connection was made between the effluent and an ESI-triple quadrupole-linear ion trap (QTRAP)-MS.

On a triple quadrupole-linear ion trap mass spectrometer (QTRAP), QTRAP® 6,500+ LC-MS/MS System, equipped with an ESI Turbo Ion-Spray interface, operating in positive ion mode, and controlled by Analyst 1.6.3 software (Sciex), linear ion trap (LIT) and triple quadrupole (QQQ) scans were obtained. The followings were the ESI source operation parameters: The ESI+ ion source, 550 °C for the source temperature, 5,500 V for the ion spray voltage, and 35 pressure for the curtain gas were all used. Multiple reaction monitoring (MRM) on a timetable was used to examine RNA alterations. Software from Sciex, Analyst 1.6.3, was used to get the data. The Sciex Multiquant 3.0.3 program was used to calculate the amounts of each metabolite. Further DP and CE optimization was done to determine the mass spectrometer’s characteristics, such as the collision energies (CE) and declustering potentials (DP), for each MRM transition. According to the metabolites eluted at each interval, a particular set of MRM transitions were observed.

### Data analysis

The statistics function prcomp in R^1^ was used to perform unsupervised principal component analysis (PCA). Prior to unsupervised PCA, the data was scaled to a unit variance. The Pearson correlation coefficients (PCC) between samples were determined by the cor function in R and given as just heatmaps, while the HCA (hierarchical cluster analysis) findings of samples and metabolites were displayed as heatmaps with dendrograms. The R package heatmap was used to do both HCA and PCC. For HCA, a color spectrum representing the normalized signal intensities of metabolites (unit variance scaling) is displayed. Absolute Log2FC (fold change) was used to identify the metabolites that significantly differed across groups. Utilizing the KEGG compound database^2^, identified metabolites were annotated. The annotated metabolites were then linked to the KEGG pathway database^3^. MSEA (metabolite sets enrichment analysis) was then run on the pathways with substantially regulated metabolites as assessed by the *P*-Values of the hypergeometric test.

### Quantitative real-time PCR

Total RNA from the indicated cells that received various treatments was extracted using the MolPure®TRIeasy Plus Total RNA Kit (19211ES60, Yeasen). Following extraction, total RNA was reverse transcribed using the All-in-one cDNA Synthesis Kit (XANCON) to create cDNA. Later, HS Universal qPCR Master Mix (BR0014-01, ACE, China) was used for qRT-PCR. The 20 μL reaction system comprised 10 μL of HS Universal qPCR Master Mix, 0.4 μL of each forward and reverse primer (10 μM), 2 μL of diluted cDNA template, and 7.2 μL of RNase-free water (Table 1). The thermocycling parameters were meticulously set asfollows: an initial denaturation at 95 °C for 30 s, followed by 40 cycles of 95 °C for 5 s and 60 °C for 30 s. A final melt curve analysis was performed to confirm primer specificity and the absence of primer dimers. The internal reference was ACTB (β-actin).

**Table 1:** Primers used in this study.

Name	Sequences (5′–3′)
GAPDH-F	TACACTGAGGACCAGGTTGTG
GAPDH-R	TTGACGAAGTGGTCGTTGAG
ALKBH1-F	CTGGTGCCCACAAGGTAGTC
ALKBH1-R	TAAGGCACTGCTTCACCCAG
ALKBH3-F	ATGCACTTCGACCGTGAGAT
ALKBH3-R	AGCAATGGCCTGGCTCTTAG
YTHDF1-F	TGCTGAAGATCATCGCCTCC
YTHDF1-R	TGTTTGTTTCGATTCTGCCGT
YTHDF2-F	CAGCCTCTTGGAGCAGAGACC
YTHDF2-R	GAAGCCAATGGAGGGACTGT
TRMT6-F	AAACCCCTGACTGAATGACCTC
TRMT6-R	ACGAGTACTGAGACACTGCC
TRMT10C-F	AAGCCAACCGGGAAGATACG
TRMT10C-R	CCTGAGTCACGGGTAACCAC

### Statistical analysis

The two-tailed Student’s *t* test was performed to compare two groups using the GraphPad Prism 5 program (GraphPad Software, United States), while a one-way analysis of variance (ANOVA) was used to compare several groups. *p*-values of less than 0.05 and 0.01 were used to determine significance levels, with ns denoting not significant. The statistics represent averages from three different tests.

## Results

### PRV growth curve and QC assessment

**Figure 1:**
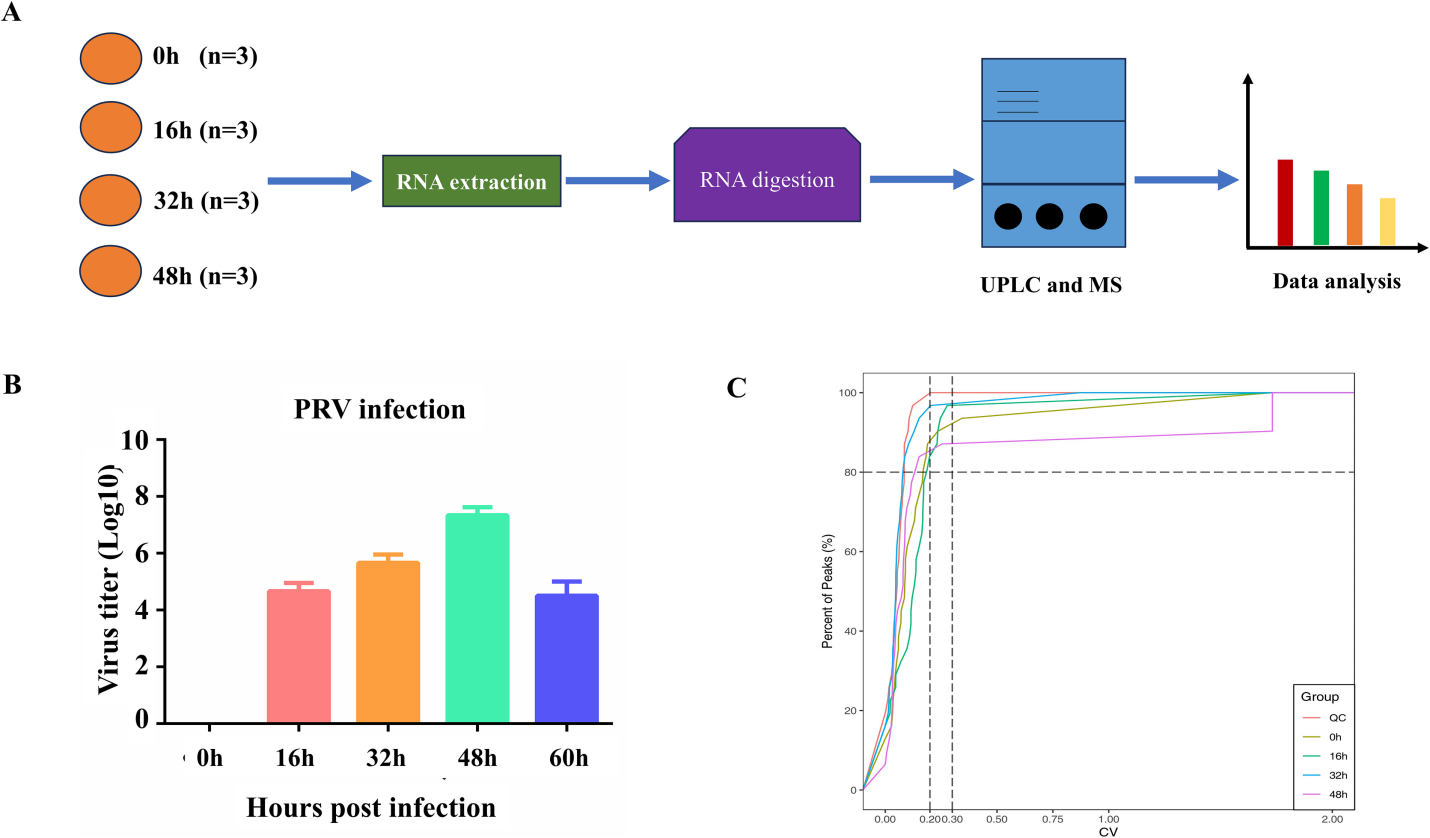
Schematic process of this study. **(A)** The overview of RNA modification analysis. **(B)** PK-15 cells were infected with PRV RA strain at a MOI of 1 and the supernatant was harvested at the indicated time periods. **(C)** QC analysis of the samples.

To acquire the understanding of RNA modifications involved in PRV infection, we performed RNA sequencing analysis in virus-infected PK-15 cells at the different time points. The schematic process was shown in Figure 1A. First, we determined the viral titer and found that it peaked at 48 h post infection (hpi) but significantly reduced at 60 hpi (Figure 1B). Additionally, the cytopathic effects of PK-15 cells were pronouncedly observed without significant occurrence of cell death until 48 hp. Next, we checked the sample quality and found that the samples were qualified for the subsequent detection, as the high proportion of substances with low coefficient of variation (CV) value in quality check (QC) samples represents the stable experimental data. In general, the proportion of substances in QC samples with CV value less than 0.3 is higher than 80%, indicating that the experimental data is stable, while the proportion of substances in QC samples with CV value less than 0.2 is higher than 80%, indicating that the experimental data is very stable. As shown in Figure 1C, the proportion of substances in the experimental samples were approaching 0.3 or 0.2, indicating they were qualified for the next step.

### Global RNA modifications were mostly reduced upon PRV infection

**Figure 2:**
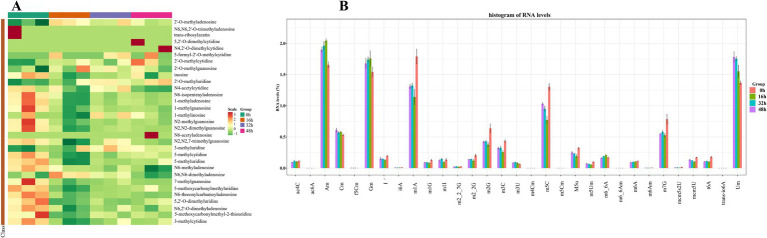
PRV infection largely suppresses the level of RNA modifications. **(A)** The heat map showed the differentially altered RNA modifications during PRV infection. **(B)** The histogram showed the alterations of different RNA modifications during PRV infection.

**Figure 3:**
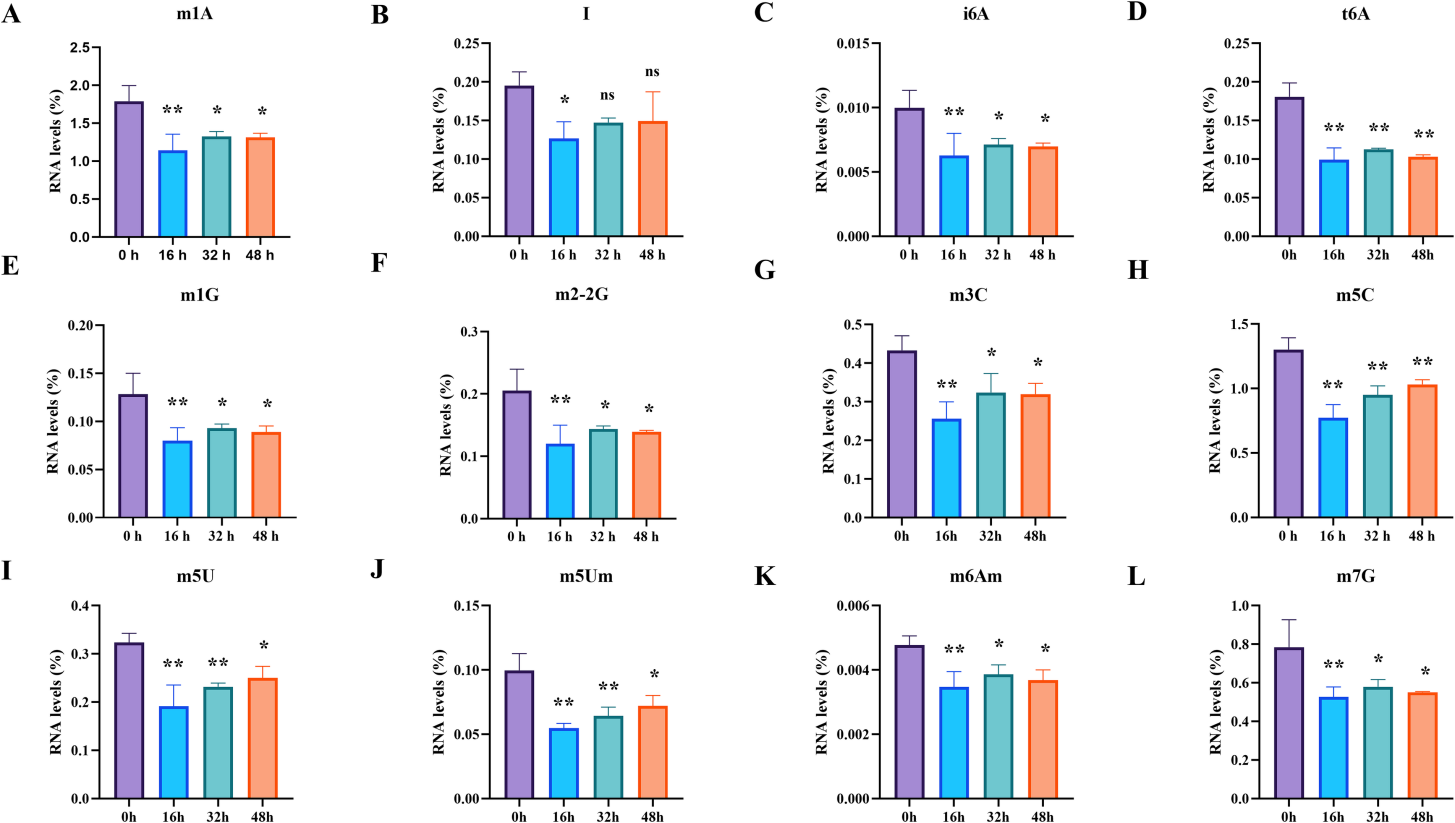
Changes in RNA modifications by PRV infection. **(A–L)** Statistical analysis of the specific RNA modification between the groups. Data are exhibited as mean ± SD. **p < 0.01; *p* < 0.05; ns, not significant.

Next, we analyzed the results obtained by the RNA sequencing. Consequently, PRV infection largely downregulated the levels of RNA modifications from both host and virus RNA (Figure 2A). Interestingly, we found that a total of 31 modifications were observed during PRV infection, with 25 modifications abundantly detected (Figure 2B), among which Am, Gm, Um, Cm, m1A, and m5C modifications were the most abundant. As shown in Figure 3, most of the observed modifications were significantly reduced, while there were several modifications increased, including Am, Cm, Gm, and Um (Supplementary Figure S1), indicating that PRV may elevate these modifications to support viral RNA, thus promoting viral replication. Many modifications can be treated as metabolites from nucleotide metabolism, such as m6_6Am, f5Cm, and mcm5s2U (Lane and Fan, 2015; Mullen and Singh, 2023). To further analyze the pathways enriched by these metabolites, we employed KEGG to map the network of possible pathways underlying altered metabolites. As shown in Supplementary Figure S2, there are multiple pathways that are enriched and regulated by the metabolites, such as aflatoxin biosynthesis, retinol metabolism, lipoic acid metabolism, sphingolipid metabolism, and ether lipid metabolism. Overall, these data suggest that upon PRV infection, most of RNA modifications are downregulated but several modifications are upregulated, which are involved in many metabolic pathways.

### The mRNA levels of m1A modification are altered during PRV infection

**Figure 4:**
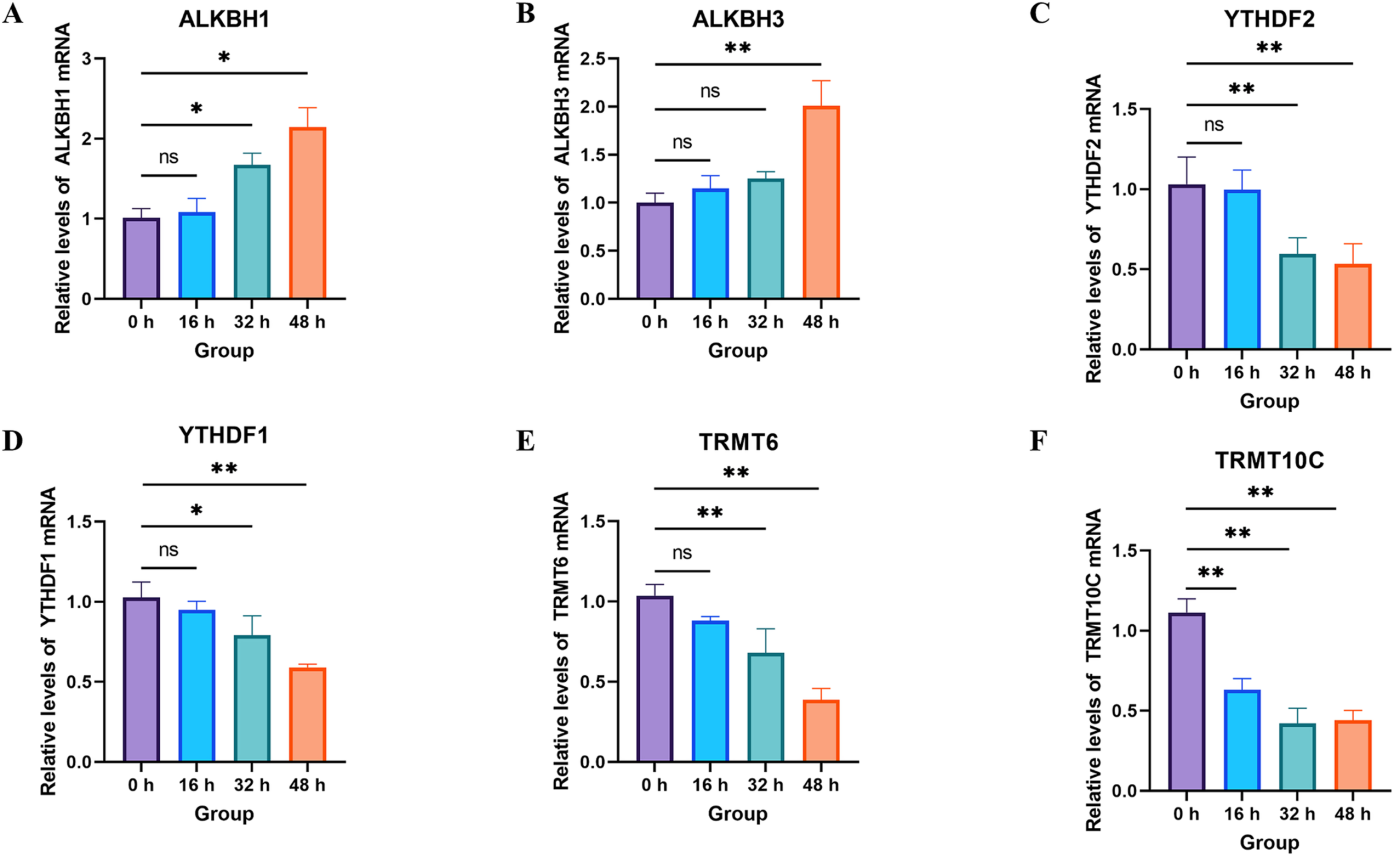
The levels of m1A modification are regulated by PRV infection. **(A–F)** PK-15 cells were infected with PRV RA strain at a MOI of 1. At the indicated time points, cells were harvested and extracted for total RNA. The mRNA levels were analyzed by RT-qPCR using the specific primers. Data are exhibited as mean ± SD. **p < 0.01; *p* < 0.05; ns, not significant.

Given that m1A was among the most abundant RNA modifications identified in our global profiling and its role in viral infections remains poorly characterized, we further investigated the expression dynamics of key m1A regulatory enzymes (erasers, readers, and writers) in PRV-infected PK-15 cells at 0 h, 16 h, 32 h, and 48 hpi via qRT-PCR (Figure 4). Consistent with the global suppression of RNA modifications, m1A regulatory enzymes exhibited coordinated expression changes: erasers ALKBH1 and ALKBH3 showed a gradual upregulation over the infection course, with significant increases at 48 hpi (**p < 0.01), while no significant differences were detected at 16 hpi and 32 hpi (ns). In contrast, m1A readers (YTHDF1, YTHDF2) and writers (TRMT6, TRMT10C) displayed a consistent downregulation, with significant reductions at 48 hpi (**p < 0.01) and no notable alterations in the early and middle infection stages. These results indicate that PRV infection induces dynamic perturbations in the m1A regulatory network, which may contribute to the global changes in RNA modification profiles observed earlier.

## Discussion

RNA modifications occur in RNA of virus and host, including coding RNA and noncoding RNA. They are known to play a role in the game between various viruses and host cells, and a thorough knowledge of RNA epigenetics will aid in the creation of antiviral medications. We found that many RNA modifications were pronouncedly reduced upon PRV infection, indicating host cells may downregulate these modifications to counteract viral replication as much as possible. At the same time, PRV may also take advantage of cellular machinery to upregulate some modifications, such as Cm, Gm, Am, and Um, to benefit its own replication. Of note, the levels of Am and Um were significantly elevated, while the levels of Cm and Gm were not significantly higher. Due to the high content of C and G in PRV genome (Pomeranz et al., 2005), it is very possible that PRV genome synthesis requires much more C and G. Because of this, C and G together with their modifications are consumed in larger amounts. Furthermore, the upregulation of 2′-O-methylated nucleosides (Am, Cm, Gm, and Um) provides a critical strategic advantage for the virus. 2′-O-methylation is a well-established mechanism utilized by various DNA and RNA viruses to modify their own mRNA cap structures, thereby structurally mimicking host mRNAs. This molecular “camouflage” allows the viral transcripts to effectively evade recognition by host innate immune sensors, such as RIG-I and MDA5. Consequently, the selective elevation of these specific modifications during PRV infection highly likely reflects the massive accumulation of viral transcripts equipped with immune-evasive 2′-O-methylation, which facilitates robust viral replication in the face of host defenses. It is important to acknowledge a technical limitation of the UPLC-MS/MS method utilized in this study. This approach measures the total cellular nucleosides from digested RNA and cannot currently distinguish whether the observed changes, such as the pronounced elevation of Am, Cm, Gm, and Um, originate from host transcripts, viral RNA, or a combination of both. Given the high abundance of viral transcripts synthesized during the late stages of PRV infection, the enriched modification signals could potentially be diluted by or directly attributed to the viral genome itself. To resolve this in future studies, techniques such as viral RNA pull-down assays coupled with LC–MS/MS, or metabolic labeling strategies (e.g., 4SU-labeling), will be essential to map these specific modifications exclusively to the PRV RNA genome.

While our time-course data (from 0 h to 48 hpi) clearly demonstrates that RNA modification landscapes shift progressively in tandem with the viral replication cycle—strongly implying a dependence on active viral synthesis—we acknowledge the absence of an ultraviolet (UV)-inactivated PRV control in our global UPLC-MS/MS screening. Future targeted studies must incorporate UV-inactivated viral controls to definitively uncouple the epitranscriptomic changes induced by active viral replication from those triggered by the initial viral attachment and entry processes.

Epitranscriptomic regulation, particularly RNA modifications such as N6-methyladenosine (m6A), 5-methylcytosine (m5C), and N1-methyladenosine (m1A), plays a pivotal role in modulating viral-host interactions (Qiu et al., 2025; Wang et al., 2025; Zhang et al., 2026). Recent studies reveal that PRV infection dynamically alters m6A levels by suppressing methyltransferases like METTL3 via viral kinase US3, thereby reducing m6A on host antiviral transcripts and impairing interferon signaling (Jansens et al., 2022). Similarly, m5C modifications influence Toll-like receptor (TLR) pathways, with enzymes such as NOP2/Sun RNA methyltransferase 5 (NSUN5) potentially methylating host mRNAs to dampen immune responses (Chen B. et al., 2023). Emerging evidence highlights m1A as a critical but understudied modification in viral pathogenesis. While m1A is predominantly found in tRNAs and rRNAs, its presence in viral RNA genomes (e.g., SARS-CoV-2) suggests a conserved mechanism where m1A incorporation disrupts RNA-dependent RNA polymerase (RdRp) activity, halting viral replication (Song et al., 2023). For PRV, m1A modifications in viral transcripts or host antiviral mRNAs could similarly may impair RdRp-mediated replication or translation. Notably, viral proteins like HSV-1 ICP0 degrade m6A writers, implying that PRV may evolve analogous strategies to manipulate m1A pathways. Therapeutically, targeting RNA modifications offers multi-pronged strategies. Restoring m6A levels via METTL3 agonists or inhibiting FTO demethylase could reinstate antiviral defenses, as demonstrated in other herpesviruses. Combining epitranscriptomic modulators with nucleoside analogs could enhance antiviral efficacy (Chen et al., 2025; Ceballos and Acevedo, 2025). Future research should prioritize mapping m1A sites in PRV RNA using advanced sequencing, elucidating viral-host enzyme interactions, and validating m1A-targeted inhibitors in animal models. Additionally, integrating multi-omics approaches could uncover synergistic targets, positioning RNA modifications as a cornerstone of next-generation antiviral therapies.

The m1A alteration was distinguished from other RNA modifications for the first time in 1961 (Zhang et al., 2023). Adenosine’s N1 position undergoes m1A alteration when a methyl group is added by m1A regulators. Three types of regulators control m1A status. They are “readers” (YTHDF1, YTHDF2, YTHDF3, and YTHDC1), “writers” (TRMT10C, TRMT61B, and TRMT6/61 A), and “erasers” (ALKBH1, ALKBH3, and FTO) (Bao et al., 2023; Li J. et al., 2022; Liu et al., 2016). In human cells, TRMT10C catalyzes the m1A modification at position 9, whereas TRMT61B and TRMT6/61 A catalyze it at position 58 in the mt- RNA (Qi et al., 2023; Chujo and Suzuki, 2012). m1A-modified sites in RNA transcripts are directly bound by proteins with YTH domains. The removal of m1A from single-stranded DNA and RNA is catalyzed by erasers, which are demethylases. It has been discovered that two proteins from the AlkB family, ALKBH3 and ALKBH1, remove m1A (Liu et al., 2016; Qi et al., 2023). The m1A modification is implicated in a number of biological processes, according to recent investigations. According to Wu et al. (2022), m1A demethylase ALKBH3 altered cancer cell glycolysis, which in turn influenced tumor development and cancer progression. Interestingly, the mRNA levels of them were significantly reduced during viral infection. This suggested that host cells may manipulate self-machinery to downregulate m1A levels as a tactic to defend against PRV infection. From this, it is promising to develop drugs targeting m1A modification-related enzymes for inhibition to treat PR. While our current study establishes the transcriptional suppression of key m1A writers and readers during PRV infection, we acknowledge that mRNA levels do not always perfectly correlate with functional protein abundance. Future investigations must prioritize confirming these regulatory shifts at the translational level via western blotting. Furthermore, to move beyond associative findings, subsequent studies should employ functional assays, such as siRNA-mediated knockdown or lentiviral overexpression of specific m1A erasers (ALKBH1/3) and readers (YTHDF)—to determine their direct impact on PRV replication kinetics. Additionally, implementing m1A-RIP-seq (RNA immunoprecipitation sequencing) will be critical for mapping the precise m1A modification sites on both host antiviral transcripts and the PRV RNA genome to elucidate the exact mechanism of virus-host epitranscriptomic interplay.

## Data Availability

The original contributions presented in the study are included in the article/Supplementary material, further inquiries can be directed to the corresponding author.

## Footnotes

1
https://www.r-project.org/

2
http://www.kegg.jp/kegg/compound/

3
http://www.kegg.jp/kegg/pathway.html

## Supplementary material

SUPPLEMENTARY FIGURE S1 Changes of RNA modifications during PRV infection. **(A–D)** Am, Um, Cm, and Gm modifications were statistical analyzed and compared. Data are exhibited as mean ± SD. ^**^*p* < 0.01; *p* < 0.05; ns, not significant.

SUPPLEMENTARY FIGURE S2 Pathway analysis at different time points (vs 0 h). **(A)** Pathway analysis of 16 h vs. 0 h; **(B)** Pathway analysis of 32 h vs 0 h; **(C)** Pathway analysis of 48 h vs. 0 h. Red indicates that metabolite levels were significantly up-regulated in the experimental group; blue indicates that metabolite levels were detected but did not change significantly; green indicates that metabolite levels were significantly down-regulated in the experimental group.
