# Supplementary figures and images for: Pseudorabies virus infection reshapes the host epitranscriptome by globally suppressing but selectively elevating specific RNA modifications
Source: Front Microbiol. 2026 Mar 27;17:1790077. doi: 10.3389/fmicb.2026.1790077 (PMC13066247; doi:10.3389/fmicb.2026.1790077)

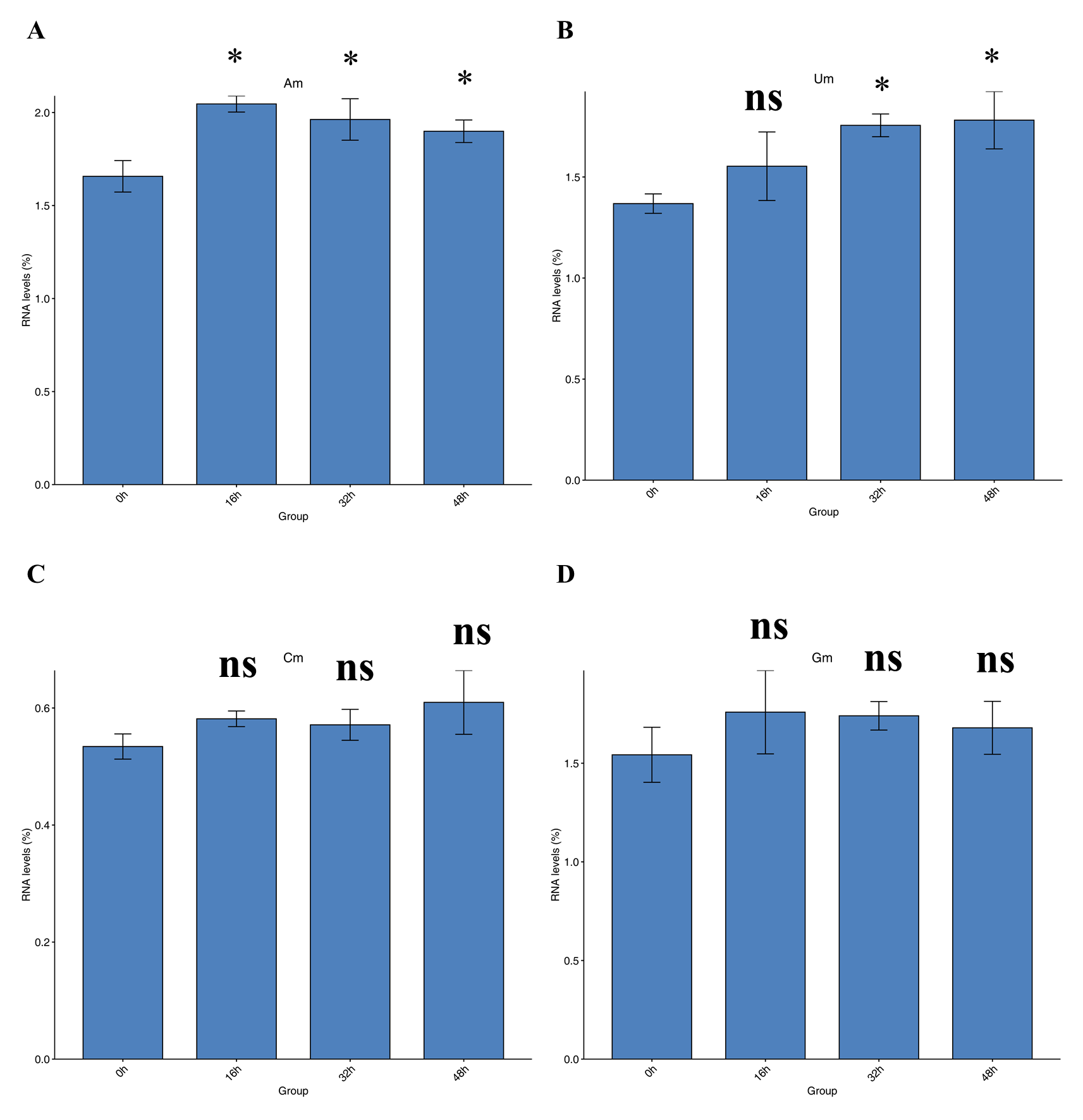

Supplement: SUPPLEMENTARY FIGURE S1 — Changes of RNA modifications during PRV infection. (A–D) Am, Um, Cm, and Gm modifications were statistical analyzed and compared. Data are exhibited as mean ± SD. **p < 0.01; p < 0.05; ns, not significant. [file Image_1.TIF]

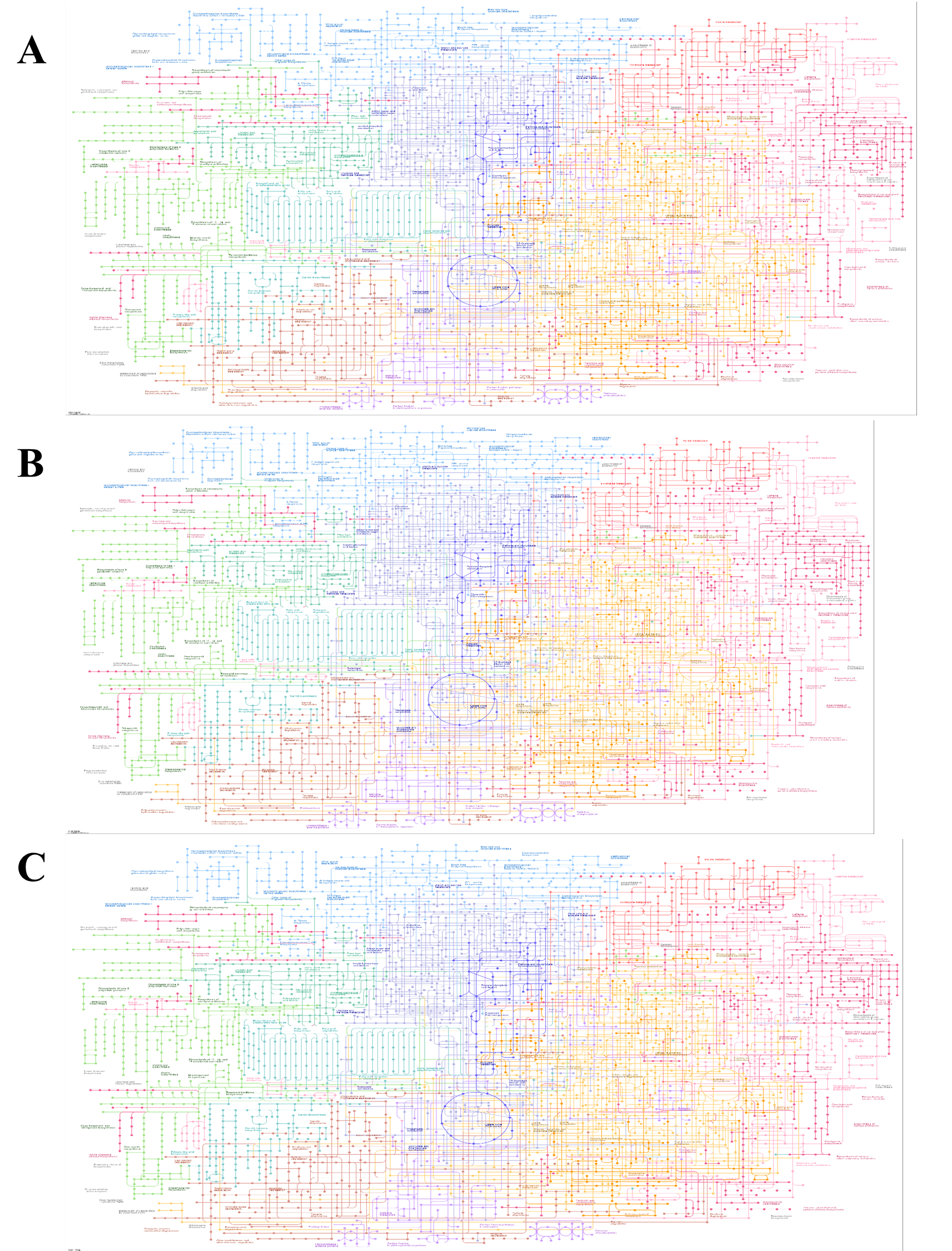

Supplement: SUPPLEMENTARY FIGURE S2 — Pathway analysis at different time points (vs 0 h). (A) Pathway analysis of 16 h vs. 0 h; (B) Pathway analysis of 32 h vs 0 h; (C) Pathway analysis of 48 h vs. 0 h. Red indicates that metabolite levels were significantly up-regulated in the experimental group; blue indicates that metabolite levels were detected but did not change significantly; green indicates that metabolite levels were significantly down-regulated in the experimental group. [file Image_2.TIF]
